# A Case of Successful Direct Oral Anticoagulant Use for the Treatment of Warfarin-Induced Vasculitis in a Patient With Left Ventricular Thrombus

**DOI:** 10.7759/cureus.50885

**Published:** 2023-12-21

**Authors:** Georgette Nader, Majid Yavari, Anisha Timilsina, Sumugdha Rayamajhi

**Affiliations:** 1 Internal Medicine, Michigan State University College of Human Medicine, East Lansing, USA; 2 Internal Medicine, Michigan State University-Sparrow Hospital, Lansing, USA

**Keywords:** warfarin-induced vasculitis, doac, vasculitis, left ventricular thrombus, leukocytoclastic vasculitis

## Abstract

Cutaneous small vessel leukocytoclastic vasculitis (LCV) is isolated to the dependent areas of the skin. LCV can be induced by pharmaceutical drugs, and management requires abrupt discontinuation of the offending drug. Warfarin is a rare medication to cause LCV, with sparse literature to date. Here, we present a case of warfarin-induced LCV, complicated by a patient’s comorbid left ventricular thrombus, and successful treatment with discontinuation of warfarin and replacement with a direct oral anticoagulant (apixaban).

## Introduction

Cutaneous small vessel leukocytoclastic vasculitis (LCV) is defined as an isolated vasculitis of the skin without systemic involvement [1]. LCV can be idiopathic or secondary to hematologic disorders, collagen vascular diseases, infections, drugs, and/or malignancies [2]. Drug-induced LCV accounts for 10-30% of cases [3]. Diagnosis is confirmed through biopsy, which shows granulocytes in perivascular or extravascular locations [4]. Treatment of LCV consists of stopping the offending agent [5].

Warfarin-induced LCV is a rare phenomenon with sparse literature. In contrast to LCV, warfarin is more commonly known to induce skin necrosis. Warfarin-induced skin necrosis is caused by blood clots that form within the blood vessels of the skin, resulting in ischemia and eventual tissue necrosis. Diagnosis is confirmed through a skin biopsy of the affected tissue [6]. Onset typically occurs within 2-10 days of warfarin therapy and affects lipid-predominant regions such as the breast, thigh, and buttocks [7].

In both warfarin-induced LCV and warfarin-induced skin necrosis, the mainstay of treatment is the prompt cessation of warfarin [2,6]. In the United States and Canada, warfarin remains the preferred anticoagulation agent in late-stage chronic kidney disease and management of left ventricular (LV) thrombus. However, upcoming trials investigating alternative anticoagulation therapy and duration are emerging [8,9]. This case describes the successful use of a direct oral anticoagulant (DOAC) (apixaban) in the treatment of apical LV thrombus, complicated by warfarin-induced LCV.

## Case presentation

**Figure 1:**
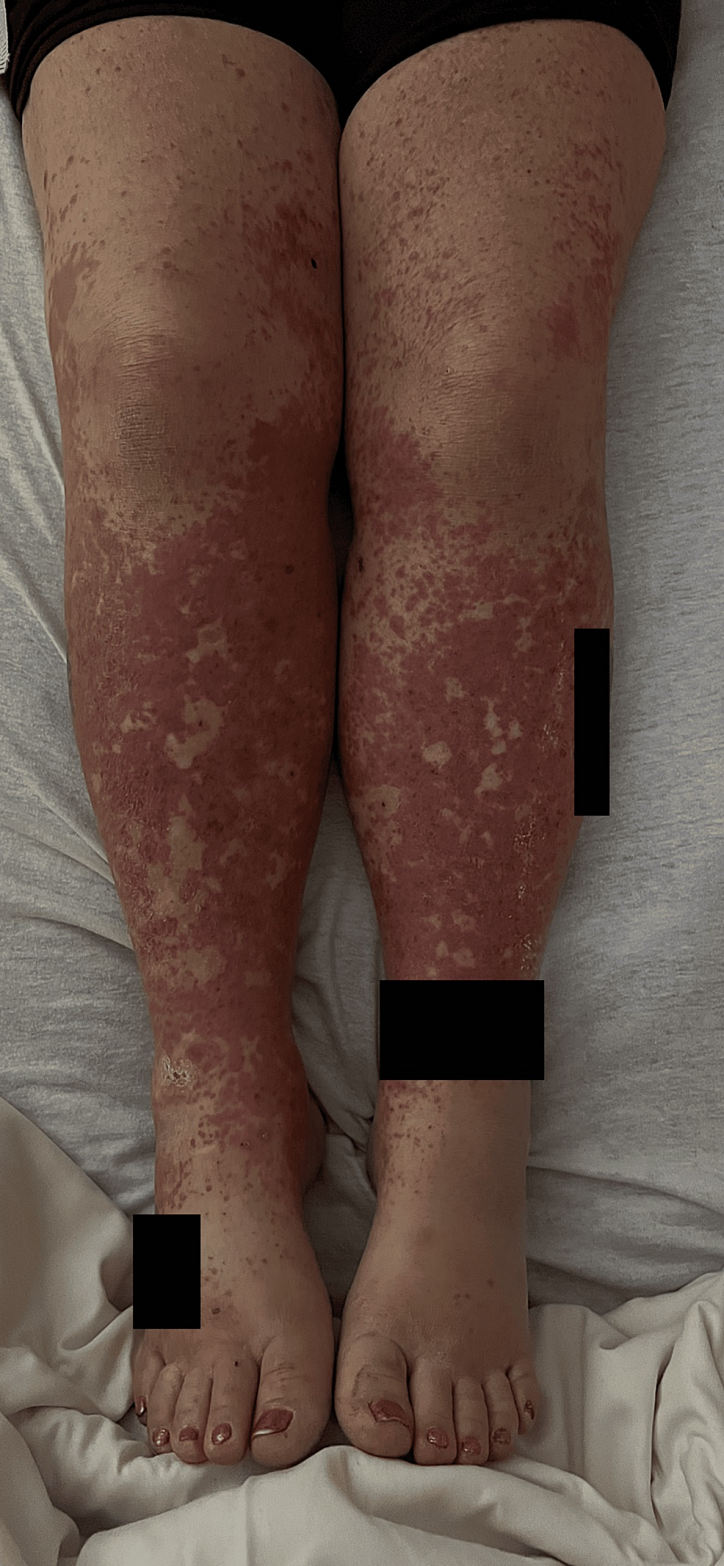
Warfarin-induced leukoclastic vasculitis pruritic rash present upon admission.

A 51-year-old female presented to the hospital with lower extremity pain and a progressively worsening rash for one month. Past medical history was significant for non-ischemic cardiomyopathy due to hypertension and polysubstance use, congestive heart failure with a reduced ejection fraction of 30-35% complicated by apical LV thrombus and multiple cardioembolic infarcts, deep vein thrombosis, peripheral vascular disease, and methicillin-resistant *Staphylococcus aureus* cellulitis. Medications included carvedilol 25 mg twice daily, furosemide 40 mg daily, hydroxyzine 25 mg daily, losartan 50 mg daily, Spironolactone 25 mg daily, and warfarin 2 mg daily which was started one month before presentation for treatment of apical LV thrombus. On presentation, the patient was vitally stable. Physical examination revealed a well-demarcated, violaceous, non-pruritic rash isolated to bilateral lower extremities (Figure 1).

The patient denied any history of a drug or food allergy, environmental or topical exposures, and/or recent lifestyle changes. The patient was admitted to the hospital. Due to high suspicion of drug-induced rash, warfarin was discontinued and the patient was started on subcutaneous low-molecular-weight heparin (LMWH) 1 mg/kg every 12 hours. Lab findings are listed in Table 1.

**Table 1:** Pertinent laboratory results.

Lab	Value obtained	Normal value
Procalcitonin	0.27 ng/mL	0–0.09 ng/mL
White blood cell count	6.3 × 103/µL	4–12 × 103/µL
Eosinophilia	3.9%	0–6%
Platelet	592 × 10^3^/µL	150–400 × 103/µL
International normalized ratio	2.1	2–3
Partial thromboplastin time	26.1 seconds	21–31 seconds
Prothrombin time	13.9 seconds	9–11.5 seconds
C-reactive protein	1.2 mg/dL	0–1 mg/dL
Erythrocyte sedimentation rate	45 mm/hour	0–15 mm/hour
Antinuclear antibody ratio	1.187	0–0.9
Double-stranded DNA	1.7 U/mL	0–9.9 U/mL
Rheumatoid factor	<20	0–20
Protein C antigen	0.94%	60–150%
C3 complement	124 mg/dL	79–152 mg/dL
C4 complement	36 mg/dL	16–38 mg/dL
Extractable nuclear antigen	<0.7 U/mL	0–0.60 U/mL
Myeloperoxidase antibody	<0.2	<0.4
Proteinase 3 antibody	<0.2	<0.4

**Figure 2:**
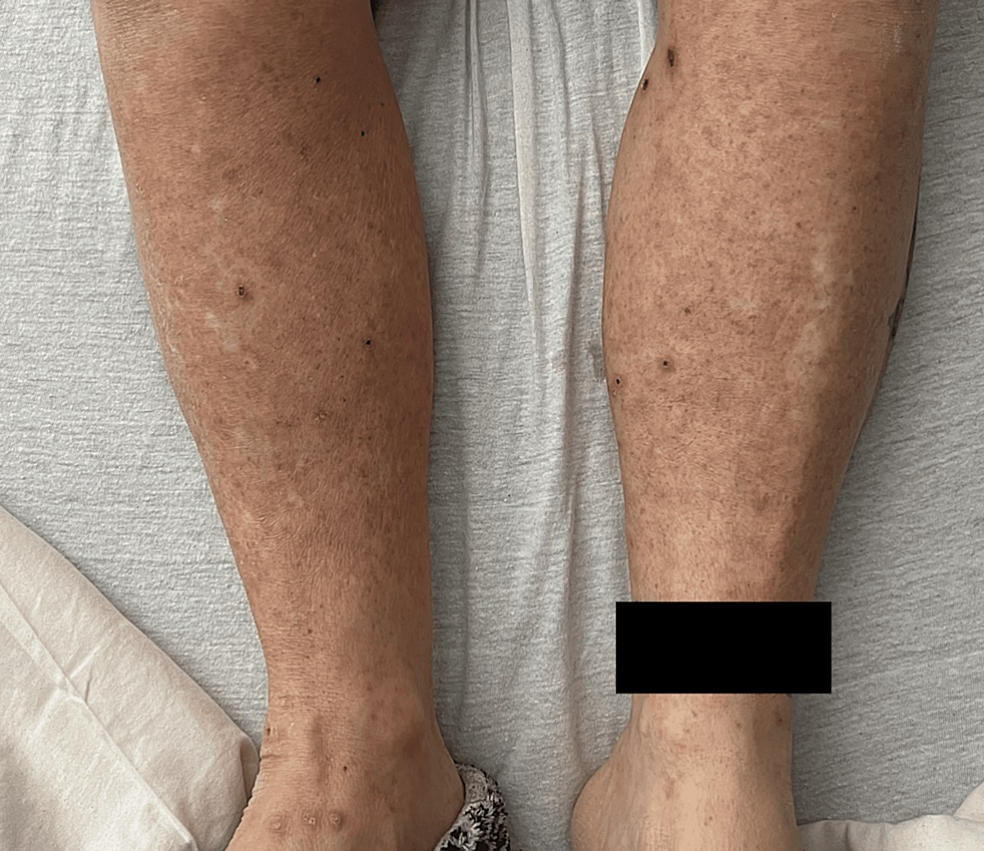
Resolution of warfarin-induced leukocytoclastic vasculitis four days after the discontinuation of warfarin.

Ultrasound was negative for lower extremity deep vein thrombosis. A skin biopsy of the rash showed a predominant perivascular neutrophilic inflammatory infiltrate, consistent with small vessel LCV (urticarial subtype). The patient’s lower extremity rash began to improve two days following the discontinuation of warfarin therapy and she had nearly complete resolution by day four (Figure 2). Corticosteroids were not initiated with such a rapid improvement upon cessation. Six weeks following hospitalization, the patient had complete resolution of the rash and had no additional rashes or thromboembolic events.

Following the diagnosis of drug-induced small vessel LCV secondary to warfarin, our team opted against the resumption of warfarin therapy. However, with the coinciding LV thrombus, anticoagulation was necessary. Despite the patient’s complicated procoagulant history, the patient refused self-injections with LMWH due to fear of needles and was placed on apixaban 5 mg twice daily.

## Discussion

As per the American College of Rheumatology, the diagnostic criteria for LCV require meeting three of the following: (1) age greater than 16 years, (2) palpable purpura, (3) initiation of medication during disease onset, (4) maculopapular rash, and (5) biopsy confirming granulocytes in a perivascular or extravascular location [4]. LCV typically begins as asymptomatic localized hemorrhages that progress to non-thrombocytopenic palpable purpura and may consist of vesicles, nodules, or hemorrhagic bullae. The rash occurs bilaterally and predominately affects the dependent areas of the body, such as the buttocks and lower extremities [10]. The timing of onset in patients with LCV typically ranges from one to three weeks [10]. Depending on the severity of presentation, typical management of LCV involves stopping the offending agent and adding a non-steroidal anti-inflammatory drug, antihistamine, corticosteroid, or immunosuppressive agent [5,10]. Management, however, becomes complicated in patients with comorbid conditions that necessitate the use of the offending drug.

Warfarin is a vitamin K antagonist commonly used to both treat and prevent arterial and venous thromboembolism. Warfarin requires frequent monitoring and consideration of diet and pharmaceutical agents. Common side effects of warfarin include photosensitivity, purple toe syndrome, and skin tissue necrosis [6,7,11]. Warfarin-induced cutaneous small vessel LCV is an uncommon but painful side effect of warfarin. Similar to other drug-induced LCV, treatment requires discontinuation of the offending drug [12].

Prompt differentiation between warfarin-induced skin necrosis and warfarin-induced LCV is crucial for proper management. Differentiating factors include the timing of onset and histopathology from a biopsy of cutaneous tissue. Warfarin-induced skin necrosis often occurs within the first few days of initiating warfarin therapy [13,14], whereas warfarin-induced LCV is more chronic and can occur weeks to months after initiation of therapy [15]. Concomitant use of warfarin and other pharmaceutical agents, such as digoxin and furosemide, has been associated with LCV [15]. Distinguishing medications based on the onset of initiation can help differentiate the pharmaceutical culprit. For instance, our patient was taking furosemide and warfarin, which can both independently cause LCV. However, as our patient was on medical therapy with furosemide for more than one year, LCV induced by furosemide was less likely than the newly introduced warfarin. Based on the timing of medication initiation, we chose to discontinue warfarin. Warfarin culpability was further supported by the rapid clinical improvement of the patient’s rash following warfarin discontinuation and despite continued furosemide therapy. Additionally, a skin biopsy is another factor of differentiation. Histopathology of a cutaneous lesion due to warfarin-induced skin necrosis displays thrombotic vasculopathy with focal epidermal necrosis in the absence of inflammation [6]. Warfarin-induced LCV is caused by perivascular or extravascular inflammation with infiltration of neutrophils, eosinophils, and basophils [16].

Complexity in management arises in patients requiring the pharmaceutical drug inducing LCV. As per the American Heart Association guidelines in 2013, vitamin K antagonists are listed as the preferred agent in patients with an LV thrombus, as studies have historically shown that patients on warfarin treatment often have complete resolution of a thrombus and are less likely to have systemic thromboembolism when compared to placebo or anti-platelet therapy [17]. This poses a challenge for patients with warfarin-induced LCV and a high risk of thromboembolism. However, recent meta-analyses have suggested that DOACs are not inferior to vitamin K antagonists and may be equally effective in patients with LV thrombus. A systematic review by Micheal et al. comparing the use of DOACs to vitamin K antagonists in the management of LV thrombus found a statistically significant decrease in stroke among patients treated with DOACs compared to vitamin K antagonists (odds ratio = 0.63, p = 0.03; I^2^ = 0%) and a reduced bleeding risk [18]. Additionally, Dalia et al. retrospectively compared eight large-scale studies comparing warfarin and DOAC use in LV thrombus and found similar effectiveness in the prevention of stroke, thromboembolic events, and the treatment of atrial fibrillation [19]. In light of recent advancements, the American Heart Association recently addressed the management of LV thrombus in the 2022 issue and has suggested that DOACs appear to be a reasonable alternative to warfarin in patients with LV thrombus after myocardial infarction, ischemic cardiomyopathy, and dilated cardiomyopathy [20]. The use of DOAC as an alternative management to warfarin may improve patient symptoms and medication compliance in patients such as ours who do not fit the above-mentioned criteria, have a high thromboembolic risk, and have comorbid LCV secondary to warfarin therapy.

## Conclusions

Small vessel LCV is a rare complication of warfarin therapy that requires prompt differentiation from warfarin-induced skin necrosis. A thorough patient history and skin biopsy are crucial for the diagnosis of warfarin-induced LCV. Management of warfarin-induced LCV may be complicated by patients’ co-morbid conditions and/or personal preferences. DOACs may be a comparable alternative to warfarin therapy in patients with high thromboembolic risk and coinciding LCV.
